# Structural basis for the DNA-binding activity of human ARID4B Tudor domain

**DOI:** 10.1016/j.jbc.2021.100506

**Published:** 2021-03-04

**Authors:** Jie Ren, Hongwei Yao, Wanhui Hu, Sarah Perrett, Weibin Gong, Yingang Feng

**Affiliations:** 1 National Laboratory of Biomacromolecules, CAS Center for Excellence in Biomacromolecules, Institute of Biophysics, Chinese Academy of Sciences, Beijing, China; 2 University of Chinese Academy of Sciences, Beijing, China; 3 Institute of Molecular Enzymology, School of Biology and Basic Medical Sciences, Soochow University, Suzhou, China; 4 CAS Key Laboratory of Biofuels, Qingdao Institute of Bioenergy and Bioprocess Technology, Chinese Academy of Sciences, Qingdao, China; 5 Shandong Provincial Key Laboratory of Synthetic Biology, Qingdao Institute of Bioenergy and Bioprocess Technology, Chinese Academy of Sciences, Qingdao, China

**Keywords:** DNA-binding protein, tumor suppressor gene, protein–DNA interaction, protein stability, protein structure, nuclear magnetic resonance (NMR), Tudor domain, protein motif, AR, androgen receptor, CSP, chemical shift perturbation, DSC, differential scanning calorimetry, EMSA, electrophoretic mobility shift assay, HSQC, heteronuclear single quantum coherence, HTD, hybrid Tudor domain, ITC, isothermal titration calorimetry, NMR, nuclear magnetic resonance, RB, retinoblastoma protein, RBBP1, retinoblastoma-binding protein 1, RMSD, root mean square deviation

## Abstract

Human ARID4A and ARID4B are homologous proteins that are important in controlling gene expression and epigenetic regulation but have distinct functions. Previous studies have shown that the N-terminal domain of ARID4A is an unusual interdigitated double Tudor domain with DNA-binding activity. However, how the Tudor domain of ARID4B differs from that of ARID4A remains unknown. Here, we found that the ARID4B Tudor domain has significantly weaker DNA affinity than the ARID4A Tudor domain despite sharing more than 80% sequence identity. Structure determination and DNA titration analysis indicated that the ARID4B Tudor domain is also an interdigitated double Tudor domain with a DNA-binding surface similar to ARID4A. We identified a residue close to the DNA-binding site of the Tudor domain that differs between ARID4A and ARID4B. The Leu50 in ARID4A is Glu50 in ARID4B, and the latter forms salt bridges with two lysine residues at the DNA-binding surface. This causes a decrease in the strength of positive charge, thus reducing DNA-binding affinity while significantly increasing protein stability. We also found that a C-terminal extension region enhances the DNA-binding affinity of the ARID4B Tudor domain. This C-terminal extension is disordered and contains a positively charged RGR motif, providing an additional DNA-binding site. Finally, sequence and phylogenetic analyses indicated that the residue differences and the presence of the RGR extension region are conserved. These results provide new insight into the functional differences between ARID4A and ARID4B proteins, as well as elucidating the function of the disordered regions in these proteins.

Human ARID4A and ARID4B, also known as retinoblastoma-binding protein 1 (RBBP1) and RBBP1-like protein 1 (RBBP1L1), are both components of the mSin3A complex, which suppresses gene expression and regulates epigenetic marks (1, 2, 3, 4, 5). Both ARID4A and ARID4B contribute to suppression of cancers such as leukemia and regulate the male reproductive process as well as the epigenetics of several genetic diseases including Prader–Willi syndrome (2, 6, 7, 8). ARID4A and ARID4B have also been identified as transcriptional coactivators for the androgen receptor (AR) and RB, which are involved in the regulation of Sertoli cell function and male fertility (9, 10). In addition to these shared functions, ARID4A and ARID4B also have important differences in their function. ARID4A contains the RB-binding motif (LXCXE), and specifically interacts with retinoblastoma protein (RB) (11), while ARID4B has no such motif. Functional studies indicate that mice heterozygous (+/−) or homozygous (−/−) for *Arid4a* deficiency were viable and fertile, while *Arid4b*^−/−^ mice were not born alive (8). Further, Sertoli cell-specific *Arid4b* knockout (*Arid4b*SCKO) mice display several unique and more severe phenotypes than *Arid4a*^−/−^*Arid4b*^+/−^ male mice (12). Searching the BioGRID database indicates that ARID4A has 40 physical and one genetic interactors, while ARID4B has 65 physical and one genetic interactors, of which 18 interactors are common for both proteins (13). Besides the fact that only ARID4A contains the RB-binding motif, the molecular basis of other functional differences between the two protein homologues is still unclear.

Both ARID4A and ARID4B contain five domains (Fig. 1*A*), three of which are Royal domains, including Tudor, PWWP, and chromobarrel domains. The other two domains are the ARID and R2 domains, which have HDAC-independent and -dependent gene repression activity, respectively. These five domains are highly conserved between the two proteins with sequence identity of 40 to 80%, while disordered regions share much lower sequence identity (<29%) (14). We have previously reported that the chromobarrel domain of ARID4A can bind to methylated histone tails and thus is responsible for the epigenetic regulation function of ARID4A (14). The Tudor domain of ARID4A was demonstrated to be an interdigitated double Tudor domain, like the Tudor domains in the three Jumonji C domain-containing histone demethylases (JMJD2A/2B/2C), in which two β-strands at the N terminus and two β-strands at the C terminus form a hybrid Tudor domain (HTD-1), and the middle four β-strands form another Tudor domain (HTD-2) (15). Tudor domains usually recognize methylated lysine or arginine (16, 17). The interdigitated double Tudor domains of JMJD2 proteins specifically bind to H3K4me3 by a conserved aromatic box in HTD-2 (18). However, the ARID4A Tudor domain does not bind to methylated histone tails due to lack of the conserved aromatic box. Instead, it binds to the DNA duplex with an affinity (*K*_D_) of 10 to 20 μM mainly *via* HTD-1 (15).

**Figure 1:**
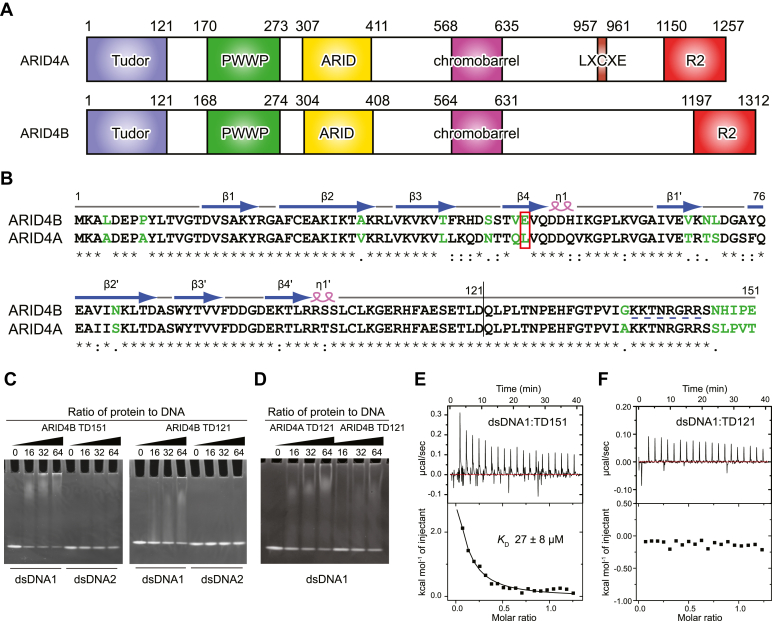
**Both the folded Tudor domain and the C-terminal tail of ARID4B TD151 bind to DNA.***A*, domain organization of ARID4A and ARID4B. *B*, sequence alignment of TD151 of ARID4B and ARID4A. The similarity of residues is shown at the *bottom*: *star* (∗), identical residue; *colon* (:), high similarity; *period* (.), low similarity; *blank*, no similarity. Residues with no or low similarity are in *green*. The secondary structure elements of ARID4B TD151 are indicated above the sequence. The *red rectangle* indicates the position of Glu50/Leu50. *C*, detection of ARID4B TD151 and TD121 interaction with DNAs by EMSA. *D*, detection of ARID4A TD121 and ARID4B TD121 interaction with dsDNA1 by EMSA. *E* and *F*, ITC titrations of dsDNA1 with ARID4B TD151 (*E*) and ARID4B TD121 (*F*).

We noticed that the region (residues 122–146) following the construct of the ARID4A Tudor domain is highly conserved between ARID4A and ARID4B with just one residue difference at position 137, and this region was predicted to possibly contain some ordered structure between residues 122 to 138 (14). This region contains five positively charged residues in the sequence 138-KKTNRGRRS-146, namely the RGR motif, which may also be a DNA-binding site. To study the function of this C-terminal disordered region, we constructed two ARID4B Tudor domain proteins containing residues 1 to 121 (TD121) and residues 1 to 151 (TD151). Surprisingly, ARID4B TD121 showed much weaker DNA-binding affinity than ARID4A TD121, although they have about 80% sequence identity. ARID4B TD151 showed a DNA-binding affinity stronger than both ARID4A TD121 and ARID4B TD121. We determined the solution structure and the DNA-binding sites of ARID4B TD151 and revealed the structural basis of the DNA-binding affinity difference. These results provide molecular insight into the functional differences between the Tudor domains of ARID4A and ARID4B and shed light on the roles of the intrinsically disordered regions in the two proteins.

## Results

### DNA-binding affinity of ARID4B Tudor domain and the role of the C-terminal extension region

We previously solved the solution structure of the ARID4A Tudor domain including residues 4 to 121, which forms an interdigitated double Tudor domain with DNA-binding activity (15). Notably, sequence containing residues 122 to 146, which follows on from the Tudor domain, is highly conserved between the two ARID4 homologues (Fig. 1*B*), implying an important function of this region. Therefore, we constructed an extended ARID4B Tudor domain containing residues 1 to 151 (TD151). We also constructed the ARID4B Tudor domain containing residues 1 to 121 (TD121) for comparison.

EMSA results showed that ARID4B TD151 has significantly stronger affinity for the 18-bp dsDNA1 than ARID4B TD121 (Fig. 1*C*), indicating that the additional C-terminal tail (residues 122–151) enhances DNA-binding affinity of the ARID4B Tudor domain. ARID4B TD151 also shows stronger affinity for the 18-bp dsDNA1 than the 12-bp dsDNA2 (Fig. 1*C*). Further, DNA-binding affinity of ARID4B TD121 is significantly weaker than ARID4A TD121 (Fig. 1*D*). Consistent with this, the affinity (*K*_D_) of ARID4B TD151 for dsDNA1 was measured as 27 ± 8 μM by ITC (Fig. 1*E*), whereas the affinity of ARID4B TD121 for DNA is undetectable (Fig. 1*F*).

### Structure determination of ARID4B TD151 and comparison with ARID4A Tudor domain

**Figure 2:**
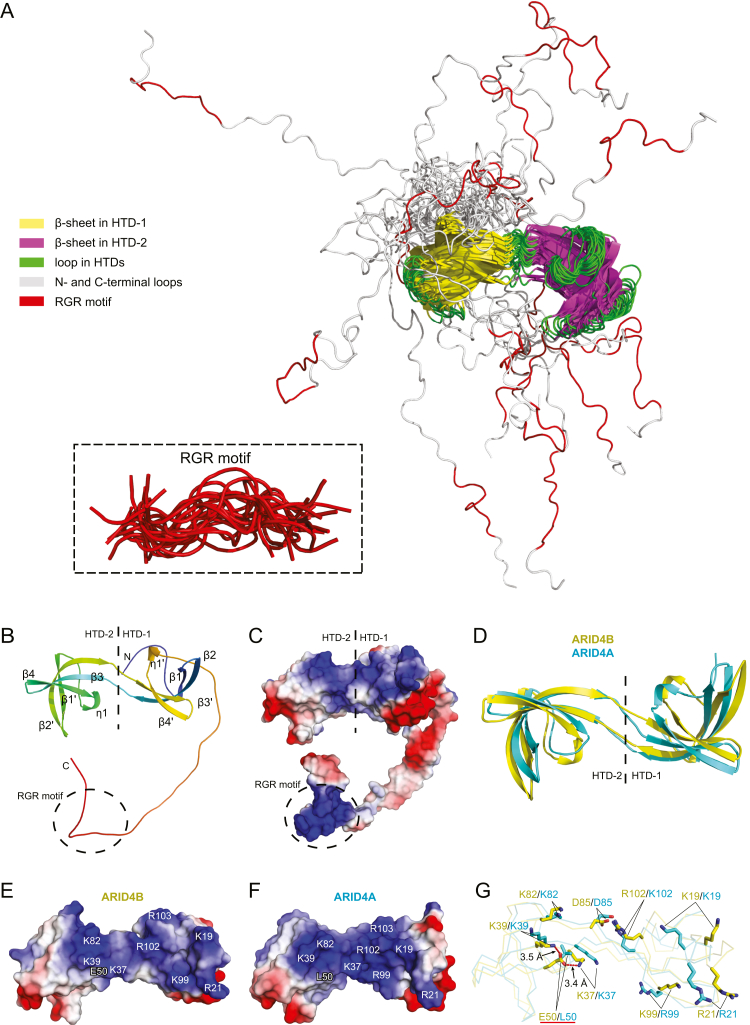
**ARID4B TD151 forms an interdigitated double Tudor domain with a 40-residue disordered C terminus.***A*, ensemble of the top 20 lowest-energy structures of the ARID4B TD151 superimposed on all secondary structure regions. The N- and C-terminal loops are disordered. The superimposed RGR motif region is shown in the *inset*. *B*, cartoon representation of the solution structure of ARID4B TD151. *C*, electrostatic surfaces of ARID4B TD151. *D*, structural superimposition of ARID4A and ARID4B Tudor domains. *E* and *F*, comparison of potential DNA-binding interfaces of ARID4B (*E*) and ARID4A (*F*) Tudor domains. The positively charged residues that may bind to DNA are labeled. *G*, comparison of charged residues at DNA-binding interfaces of the two Tudor domains.

To investigate the structural basis of the DNA-binding affinity of ARID4B TD151, we first assigned the ^1^H-, ^15^N-, and ^13^C-NMR resonances of ARID4B TD151. The ^1^H-^15^N HSQC spectrum of ARID4B TD151 and the assignments of the amide signals are shown in Fig. S1*A*. All nonproline backbone amide proton and nitrogen signals of TD151 were assigned, except for His148. The assignment of the backbone resonances (N, H^N^, C^α^, H^α^, and C') of residues Met1–Glu151 was completed to 99.4%. Assignment of the aliphatic and aromatic side chains was achieved to 94.1% (side chain amino and guanidine group atoms of lysine and arginine residues, OH, SH, side chain ^13^C', ^13^C^ξ^, and quaternary ^13^C were excluded). Based on the assignments, we then determined the solution structure of ARID4B TD151 (Table 1, Fig. 2, *A*–*C*, and Fig. S2). Like the Tudor domain of ARID4A, residues 8 to 110 of ARID4B TD151 also form an interdigitated double Tudor domain structure, containing two subdomains (hybrid Tudor domains, HTDs), HTD-1 and HTD-2. The N- and C-terminal loops of ARID4B TD151 are disordered and do not interact with the HTDs (Fig. S2*A*). HTD-1 contains four β-strands, *i.e.*, β1 (residues 15–20), β2 (residues 23–33), β3' (residues 88–93), and β4' (residues 96–101). Arg103-Ser105 forms a 3_10_ helix (η1') in some conformers. HTD-2 also contains four β-strands, *i.e.*, β3 (residues 36–42), β4 (residues 47–52), β1' (residues 63–69), and β2' (residues 74–85). Asp53-His55 also forms a 3_10_ helix (η1) in some conformers. The root mean square deviation (RMSD) values of secondary backbone atoms of HTD-1, HTD-2, and both HTDs are 0.51 Å, 0.36 Å, and 2.22 Å, respectively (Fig. S2, *A*–*C*, and Table 1). Although previous predictions of secondary structure and disordered regions indicated that sequence containing residues 122 to 138 of ARID4A possibly forms some ordered structure (14), the structure determination, CSI analysis (19), and TALOS-N results all show that residues 110 to 151 are in fact in a disordered conformation (Fig. S1, *B* and *C*, and Fig. 2*A*).

**Table 1:** The experimental restraints and structural statistics for the 20 lowest-energy conformers of ARID4B TD151

Distance restraints		
Intraresidue	1016	
Sequential	631	
Medium	248	
Long-range	764	
Ambiguous	1061	
Total	3720	
Hydrogen bond restraints	37	
Dihedral angle restraints		
Φ	85	
Ψ	85	
Total	170	
Violations		
NOE violations (>0.3 Å)	0	
Torsion angle violation (>5°)	0	
PROCHECK statistics^a^ (%)		
Most favored regions	92.2	
Additional allowed regions	6.8	
Generously allowed regions	0.2	
Disallowed regions	0.8	
RMSD from mean structure (Å)	HTD-1	HTD-2
Backbone heavy atoms		
All residue^b^	0.83 ± 0.11	0.52 ± 0.12
Regular secondary structure^c^	0.51 ± 0.08	0.36 ± 0.10
All heavy atoms		
All residue	1.31 ± 0.13	0.96 ± 0.12
Regular secondary structure	1.08 ± 0.13	0.86 ± 0.14
Backbone RMSD of all secondary structure	2.22 ± 1.13	

a PROCHECK analysis calculated parameters of residues 8 to 110 of ARID4B.

b All residues includes residues 8 to 34 and 87 to 110 for HTD-1; and residues 35 to 86 for HTD-2.

c Residues of secondary structure includes residues 15 to 20, 23 to 33, 88 to 92, and 98 to 108 of HTD-1; and residues 36 to 42, 48 to 57, 65 to 69, and 75 to 85 for HTD-2.

The structure of the ARID4B Tudor domain is highly similar to the ARID4A Tudor domain, with RMSD 0.66, 0.77, and 2.0 Å between secondary structure backbone atoms of the two HTD-1, the two HTD-2, and the two overall structures, respectively (Fig. 2*D* and Fig. S2*D*). However, compared with the positively charged DNA-binding interface of the ARID4A Tudor domain, the positive charge of the corresponding surface regions of the ARID4B Tudor domain is significantly reduced (Fig. 2, *E* and *F*). Detailed analysis indicates that the ARID4B Tudor domain contains a negatively charged residue, Glu50, at the interface, corresponding to Leu50 in the ARID4A Tudor domain, while the other charged residues in the interface are conserved between the Tudor domains of ARID4A and ARID4B (Fig. 2*G*). The carboxyl group of the Glu50 side chain forms two salt bridges with the side chain amino groups of Lys37 and Lys39. Distances between the O^ε^ atom of Glu50 and the N^ξ^ atoms of the two lysine residues are 3.4 ± 1.0 and 3.5± 1.8 Å, respectively, forming two strong salt bridges (Fig. 2*G*).

### Both the interdigitated double Tudor domain and the C-terminal RGR motif of ARID4B TD151 interact with DNA

**Figure 3:**
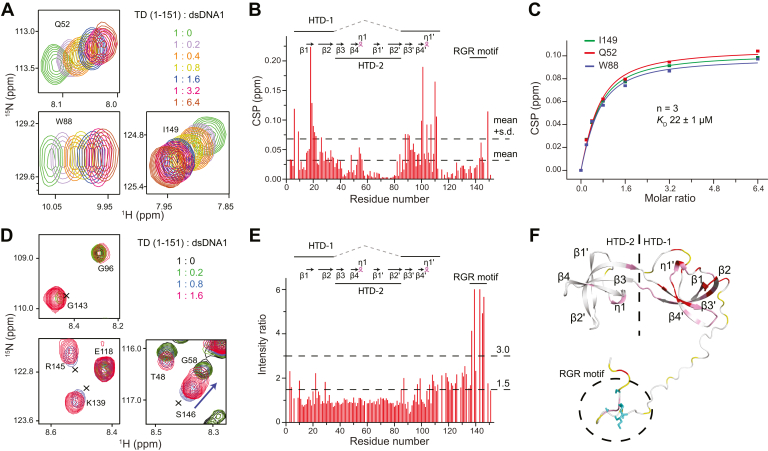
**Two DNA-binding sites of ARID4B TD151 detected by NMR titration.***A*, detection of ARID4B TD151 interaction with dsDNA1 by NMR. *B*, CSP of ARID4B TD151 *versus* residue number. *C*, affinity (*K*_D_) obtained from the fit. *D*, signal enhancements of C-terminal residues of ARID4B TD151 due to DNA addition. *E*, intensity ratio of ARID4B TD151 in the presence or the absence of dsDNA1 at a ratio of protein:DNA of 1.0:1.6. *F*, mapping of CSP and intensity ratio results onto ARID4B TD151 structure. *Red*, CSP ≥ mean + S.D.; *pink*, mean + S.D. > CSP ≥ mean; *white*, CSP < mean; *yellow*, prolines and residues without backbone NH signal assignment or with NH signals severely overlapped. The side chains of residues with an intensity ratio >3.0 are shown as *cyan sticks*.

We then performed NMR titration of ARID4B TD151 by gradually adding dsDNA1, which caused significant chemical shift perturbations (Fig. 3, *A* and *B*). The affinity was obtained by fitting the NMR titration data and the *K*_D_ obtained was 22 μM (Fig. 3*C*), in agreement with the ITC result. Besides chemical shift perturbations, addition of dsDNA1 also enhanced NH signal intensities of C-terminal disordered residues of ARID4B TD151, mainly within the sequence 137-GKKTNRGRRS-146 (RGR motif, intensity ratio >3) and Gly110-Ile136 (3 > intensity ratio >1.5) (Fig. 3, *D* and *E*). This suggests that the RGR motif of ARID4B TD151 undergoes intermediate exchange between multiple conformations in the free protein leading to line broadening, but adopts a more rigid conformation with stronger and sharper signals when bound to dsDNA.

We then mapped the results of CSP and intensity enhancement onto the structure of ARID4B TD151. Residues showing the largest CSP (colored red in Fig. 3*F*) include Glu6, Tyr9, Ala18-Gly22, Glu26, Gln52^sc^, Trp88, Thr90-Val91, Thr100-Leu101, Ser104, Cys107, Gly110-Glu111, and Ile149; residues showing a moderate CSP (colored pink in Fig. 3*F*) include Val16-Ser17, Cys25, Ala27, Ile29, Thr31, Lys33-Leu35, Thr48, Asp54-His55, Thr84, Val92-Asp95, Asp97-Glu98, Arg102-Arg103, Ser105, Lys109, and Gly143-Arg145. These residues are mainly located within HTD-1, η1 of HTD-2, and the C-terminal RGR motif (Fig. 3*F*), indicating important roles of these residues in DNA interaction. Residues showing the largest intensity enhancement (intensity ratio > 3) include Gly137, Lys139, Gly143, Arg145, and Ser146, which are all located in the C-terminal RGR motif (cyan sticks in Fig. 3*F*). In summary, ARID4B TD151 contains two DNA-binding sites. The first DNA-binding site is mainly located within HTD-1 of the interdigitated double Tudor domain, similar to the ARID4A Tudor domain (15), while the second is the RGR motif containing a heavily positively charged region, which may contribute to DNA binding by electrostatic interactions (Fig. 3*F*).

**Figure 4:**
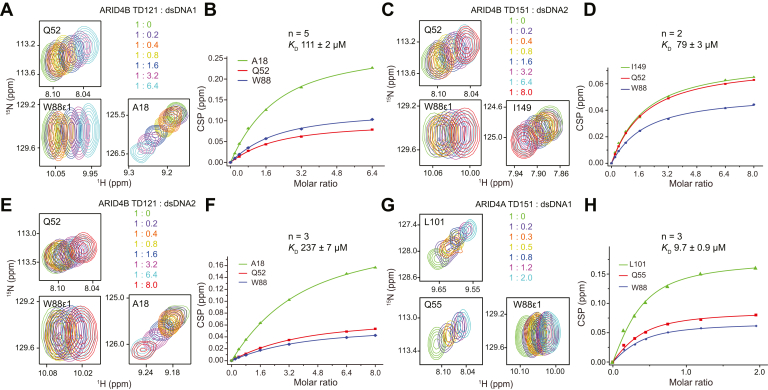
**Effect of length of DNA duplex and protein C-terminal disordered region on DNA-binding affinity of ARID4B Tudor domain measured by NMR titration.***A* and *B*, titration of ARID4B TD121 with dsDNA1 (*A*) and the affinity obtained from the fit (*B*). *C* and *D*, titration of ARID4B TD151 with dsDNA2 (*C*) and the affinity (*D*). *E* and *F*, titration of ARID4B TD121 with dsDNA2 (*E*) and the affinity (*F*). *G* and *H*, titration of ARID4A TD151 with dsDNA1 (*G*) and the affinity (*H*).

ARID4B TD121 has an affinity (*K*_D_) of ∼110 μM for dsDNA1 estimated by fitting the NMR titration data (Fig. 4, *A* and *B*), about five times weaker than ARID4B TD151, which is consistent with the EMSA result. Similarly, the affinity of ARID4B TD121 for dsDNA2 obtained from titration was 237 μM, about three times weaker than ARID4B TD151 (*K*_D_ 79 μM) (Fig. 4, *C*–*F*). Consistent with this, the affinity of ARID4A TD151 with dsDNA1 was measured as 9.7 μM (*K*_D_) (Fig. 4, *G* and *H*), about 2 to 3 times stronger than ARID4A TD121 (*K*_D_ 27 μM) (15) and ARID4B TD151 (*K*_D_ 22 μM). These results confirm that the C-terminal disordered region can enhance DNA-binding affinity of the Tudor domain.

### RGR motif of ARID4B TD151 weakly prefers AT-rich DNA

**Figure 5:**
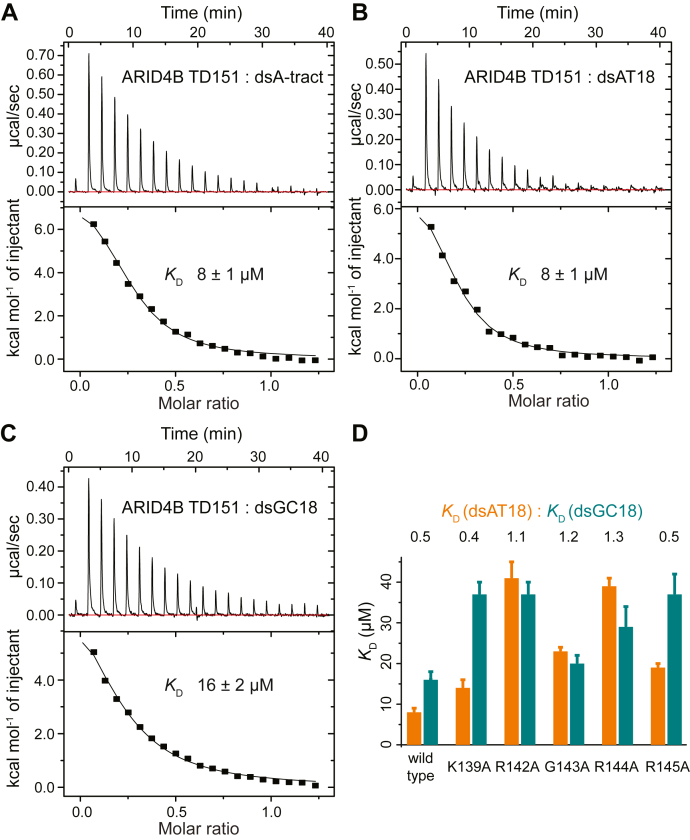
**RGR motif of ARID4B TD151 weakly prefers AT-rich DNA.***A*–*C*, ITC titrations of dsA-tract, dsAT18, and dsGC18 with ARID4B TD151. *D*, the equilibrium dissociation constants of the RGR motif mutants with AT- and GC-rich dsDNAs obtained by ITC measurements.

The C-terminal RGR motif of ARID4B TD151 is similar to the AT-hook motif, which contains a Pro-Arg-Gly-Arg-Pro sequence (20). The RGR motif of ARID4B TD151 lacks the two proline residues. As the AT-hook motif is well known to specifically interact with AT-rich DNA, especially with A-tract DNA (21), we measured affinities of ARID4B TD151 for AT-rich, A-tract, and GC-rich DNAs, finding that AT-rich and A-tract DNAs have similar affinities, while both are about twofold stronger than GC-rich DNA (Fig. 5, *A*–*C*), suggesting a very weak sequence preference for AT-rich DNA. To elucidate the role of each residue of the RGR motif in DNA binding, we constructed a series of point mutants in the C-terminal RGR motif of ARID4B TD151, including K139A, R142A, G143A, R144A, and R145A, and measured their binding affinity with dsAT18 and dsGC18 (Fig. 5*D*). The results showed that these mutations decreased the affinity by up to fivefold. Affinity ratios of dsAT18 *versus* dsGC18 binding to mutants R142A, G143A and R144A, which are core residues in the RGR motif, are ∼1.1 to 1.3, while the ratios for the mutants K139A and R145A are ∼0.4 to 0.5, which is similar to wild-type ARID4B TD151 (ratio 0.5) (Fig. 5*D*). These results indicated that the weak preference of ARID4B TD151 for AT-rich DNA is achieved by the core residues Arg142, Gly143, and Arg144, while surrounding residues such as K139 and R145 do not contribute to the preference but contribute to the overall DNA-binding affinity.

### Glu50 decreases DNA affinity but increases thermostability of ARID4B TD121

**Figure 6:**
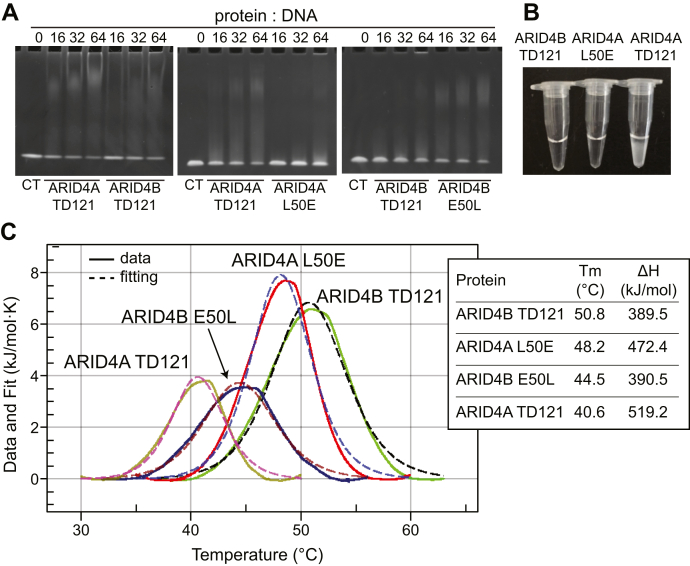
**Effect of different residues at position 50 on the DNA-binding affinity and protein thermostability of the ARID4A and ARID4B Tudor domains.***A*, detection of DNA interaction with ARID4A TD121 and its L50E mutant and with ARID4B TD121 and its E50L mutant by EMSA. CT, dsDNA1 alone. The *left panel* is the same image as in Figure 1*D* for comparison. *B*, stability test for ARID4B TD121, ARID4A TD121, and its L50E mutant at 30 °C. *C*, DSC results for TD121 of ARID4A and ARID4B and their position 50 mutants, as indicated.

Previous studies have obtained *K*_D_ values for binding of ARID4A TD121 to dsDNA1 and dsDNA2 of 27 μM and 16 μM, respectively (15), which are 4 to 15 times stronger than the affinities of ARID4B TD121 for these dsDNAs (Fig. 4, *A*, *B*, *E* and *F*). As stated above, the structure determination revealed that most of the charged residues of the DNA-binding site of ARID4A Tudor domain are conserved in the ARID4B Tudor domain except that Leu50 is replaced by the negatively charged Glu50 in the ARID4B Tudor domain (Fig. 2*G*). Glu50 in the ARID4B Tudor domain forms two salt bridges with Lys37 and Lys39, while the corresponding residues in the ARID4A Tudor domain are indicated to contact with DNA (15). We therefore suspected that Glu50 is the key reason for the lower DNA-binding affinity of the ARID4B Tudor domain. To confirm the effect of Glu50/Leu50 on DNA binding, we mutated Glu50 of ARID4B TD121 to Leu, and Leu50 of ARID4A TD121 to Glu. EMSA results showed that the E50L mutation of ARID4B TD121 increased the DNA-binding affinity, while the L50E mutation of ARID4A TD121 decreased the DNA-binding affinity (Fig. 6*A*), confirming the attenuation effect of Glu50 on DNA binding. Interestingly, this mutation significantly increased the stability of the ARID4A Tudor domain, as the mutant did not precipitate after 2 h incubation at 30 °C, similar to ARID4B TD121, while the wild type underwent significant precipitation under the same conditions, with only ∼30% protein remaining in the supernatant (Fig. 6*B*). The effect of Glu50/Leu50 on the stability was further investigated by DSC, which indicated that the L50E mutation of ARID4A TD121 increased the *T*_m_ value from 40.6 °C to 48.2 °C, while the E50L mutation of ARID4B TD121 decreased the *T*_m_ value from 50.8 °C to 44.5 °C (Fig. 6*C*). Therefore, the salt bridges between Glu50 and Lys37/Lys39 in the ARID4B Tudor domain significantly increase thermostability of the protein and simultaneously decrease its DNA-binding affinity.

### HADDOCK structure model of ARID4B TD151 complex with DNA

Based on the NMR titration results for ARID4B TD151 using dsDNA1, we attempted to construct a structural model for the complex of ARID4B TD151 and dsDNA1 using HADDOCK docking (22). The chemical shift perturbations obtained from the NMR titration experiments were used to define residues involved in the interaction, which includes residues from the folded region and the C-terminal segment, 138-KKTNRGRRS-146 and Ile149. However, we found that the long C-terminal loop between Gly110 and Gly137 disturbed model construction if we used the full-length ARID4B TD151 in the docking. We therefore used the structural region (residues 9–110) and the C-terminal peptide (138-KKTNRGRRS-146) separately for HADDOCK docking.

**Figure 7:**
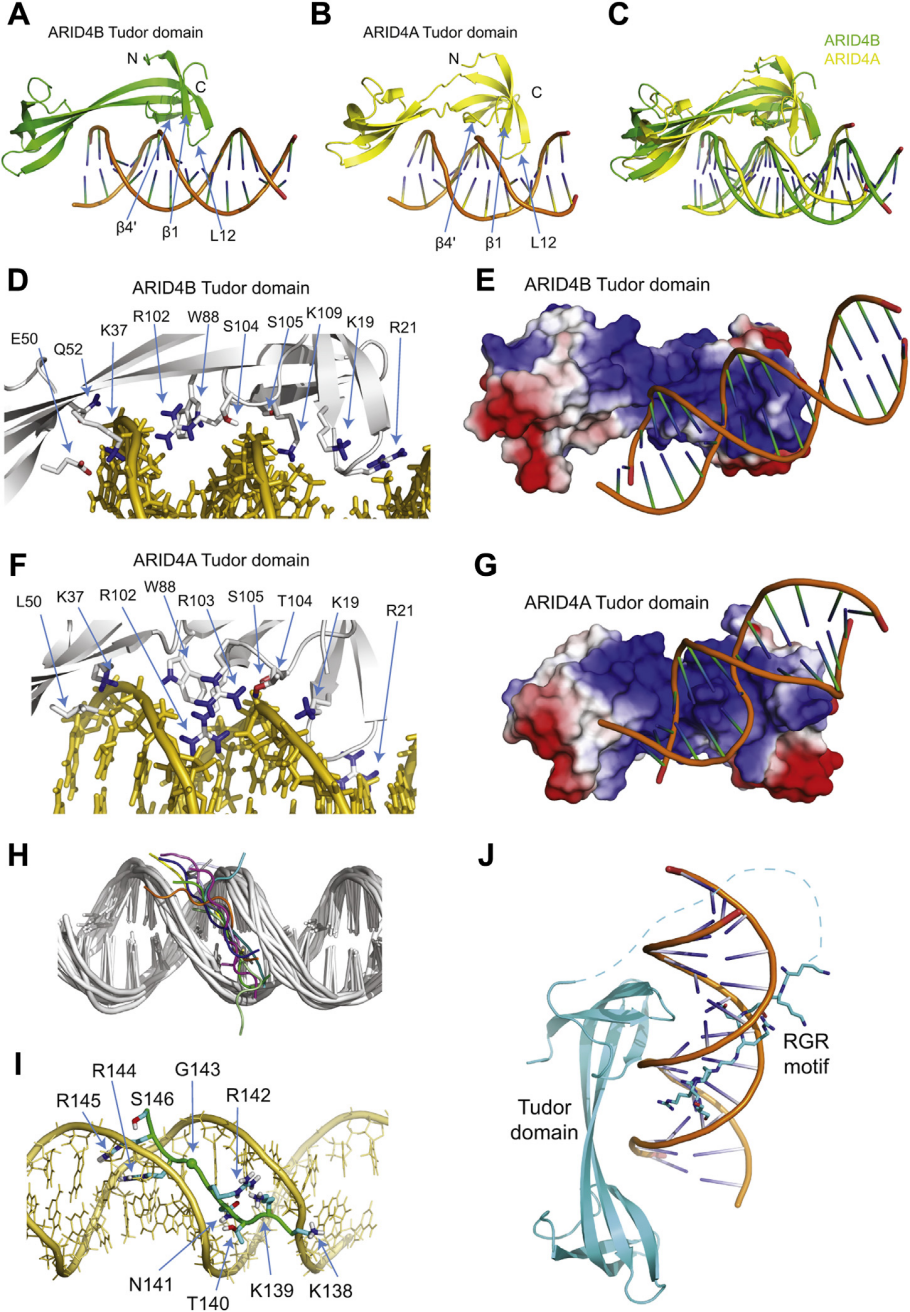
**Structural model of the complex of ARID4B TD151 with DNA**. The model was obtained using HADDOCK (22). *A*, representative structure model of the complex of ARID4B Tudor domain and dsDNA1. *B*, structure model of the complex of ARID4A Tudor domain and dsDNA2 obtained in a previous study (15). *C*, alignment of the two structures shown in *A* and *B*. *D* and *E*, contact residues (*D*) and interacting surface (*E*) of ARID4B Tudor domain and the DNA duplex in the complex. *F* and *G*, contact residues (*F*) and interacting surface (*G*) of ARID4A Tudor domain and the DNA duplex in the complex. Residues Glu50 of ARID4B and Leu50 of ARID4A are also shown in *D* and *F*. *H*, cartoon view of representative ten structures with lowest HADDOCK scores of TD151 C-terminal peptide, 138-KKTNRGRRS-146, and dsDNA1. *I*, contacts between the peptide and dsDNA1. *J*, structure model of the complex of ARID4B TD151 and dsDNA1 by manually combining the models in figure (*A*) and (*H*). It is worth noting that the combined model is not a docking result using full-length TD151 and thus the distance and orientation between the Tudor domain and RGR motif in this model are arbitrarily chosen without further optimization.

Analysis of the final 200 HADDOCK models for the ARID4B Tudor domain and dsDNA1 complex resulted in ten clusters and the statistics of the top seven clusters are displayed in Table S1. The top cluster has the largest number of structures and the lowest HADDOCK- and Z-scores, with an RMSD value of 2.0 ± 1.2 Å and buried surface area of 1422 ± 161 Å^2^. The best model in the top cluster is shown in Figure 7*A*.

In this model of the complex, the DNA duplex mainly binds to the ARID4B HTD-1 at sites containing β1, β4', loop L12, the C-terminal loop of HTD-1, and the linker between HTD-1 and HTD-2 (Fig. 7*A*). The axis along HTD-1 and HTD-2 of TD151 is almost parallel to the DNA duplex, with L12 and β1 making contact with the DNA major groove, while β4' and the C-terminal loop of HTD-1 make contact with the DNA minor groove and backbone atoms. The structure model of the complex is quite similar to that of the ARID4A Tudor domain with dsDNA2 (Fig. 7, *B* and *C*). Detailed analysis of the model indicates that residues Lys19–Arg21 of L12 extend into the DNA major groove, whereas the side chains of Trp88 and Arg102 of the C terminus extend into the minor groove (Fig. 7*D*). Side chains of Lys37 and Gln52 in HTD-2, and Ser104 and Ser105 in HTD-1 also contact the DNA backbone atoms, while Glu50 has no contact with DNA but still forms a salt bridge with Lys37 (Fig. 7*D*). These residues form a positively charged DNA-binding surface, which has electrostatic interactions and physical complementarity with DNA (Fig. 7*E*). These contact residues and the positively charged interacting surface of the ARID4B Tudor domain are basically similar to the structure model of the complex between ARID4A Tudor domain and DNA, although the positive charge of ARID4B Tudor domain is partially neutralized by Glu50 (Fig. 7, *F* and *G*).

We then built the HADDOCK model of the DNA complex with the C-terminal RGR motif (138-KKTNRGRRS-146) of ARID4B TD151. As the short segment of peptide is not suitable for cluster analysis using HADDOCK, we chose ten structures with the lowest HADDOCK scores from the final 200 structures to represent the complex in the model (Fig. 7*H*). In all ten structures, the peptide binds to the DNA minor groove. In the representative structural model, side chains of N141, R144, and R145 penetrate into the groove, and the positively charged side chains of lysine and arginine residues also have electrostatic interaction with DNA (Fig. 7*I*). Combining the HADDOCK-derived structures of the complexes of the ARID4B Tudor domain and the C-terminal RGR peptide with DNA, Figure 7*J* shows a model in which the ARID4B Tudor domain and the C-terminal peptide cooperatively bind to the DNA duplex. The Tudor domain binds to both major and minor grooves of the DNA duplex, while the RGR motif only binds to the minor groove. By sliding and rotation along the grooves of the dsDNA1, binding of about three ARID4B TD151 molecules could be accommodated (Fig. S3), which agrees with the stoichiometry obtained in NMR and ITC experiments. Interestingly, the model also indicates that the structured Tudor domain and the RGR motif can bind to opposite sides of a short DNA duplex without spatial hindrance, because the linker between the Tudor domain and the RGR motif is around 30 residues, which is long enough to allow binding to distal sites.

## Discussion

This study reveals that the Tudor domains of ARID4A and ARID4B have different DNA-binding affinities and stability, although the two Tudor domains share ∼80% sequence identity. Our results indicate that both domains bind to DNA using similar structural regions, but the one-residue difference at position 50 is the major reason for the differences in DNA-binding affinity and protein stability. Interestingly, detailed structure-based alignment of the two Tudor domains indicates that most residues that differ between the two Tudor domains (green residues in Fig. 1*C*) are located either within HTD-2 (Fig. S4*A*) or in the N- and C-terminal disordered regions. These residues are generally far away from DNA-binding sites, except that Glu50 of ARID4B forms two salt bridges with DNA-binding lysine residues and decreases the DNA affinity. Besides Glu50, we noticed that a hydrophobic residue Val68 is located in the highly hydrophobic core of HTD-2 of the ARID4B Tudor domain, surrounded by hydrophobic residues Phe42, Val49, and Val51 (Fig. S4*B*), while in the ARID4A Tudor domain structure, the corresponding residue is a less hydrophobic Thr68 surrounded by Leu42, Gln49, and Val51 (Fig. S4*C*). Because both Val68 of ARID4A and Thr68 of ARID4B are in the hydrophobic core, the difference in their hydrophobicity may also lead to the observed difference in stability. Consistent with this, we found that wild-type ARID4B TD121 (*T*_m_ 50.8 °C) has greater thermostability than the L50E mutant of ARID4A TD121 (*T*_m_ 48.2 °C), and the E50L mutant of ARID4B TD121 (*T*_m_ 44.5 °C) has greater thermostability than wild-type ARID4A TD121 (*T*_m_ 40.6 °C) (Fig. 6*C*), suggesting an important role of hydrophobic core formation involving Val68 and surrounded residues for stability of the interdigitated Tudor domain.

Besides the core interdigitated double Tudor domain, we also investigated the C-terminal positively charged disordered RGR motif of ARID4B TD151 and found that the RGR motif can bind to the DNA duplex and enhance the DNA-binding affinity of ARID4B TD151 by about fivefold. The docking results indicate that the C-terminal RGR motif prefers to bind to the DNA minor groove. The RGR motif is similar to the AT-hook motif containing a conserved Arg-Gly-Arg-Pro sequence, which can penetrate into the minor groove through the side chains of the two Arg residues during DNA binding (20). However, the RGR motif of ARID4B lacks the proline residue, which is conserved in the AT-hook motif and proposed to be critical for conformational adaption of the AT-hook motif to the DNA minor groove (20). Our results indicate that the lack of the two prolines leads to a very weak preference of ARID4B RGR motif for AT-rich DNA, which we confirmed by mutational analysis. Therefore, the RGR motif can be considered as an AT-hook-like motif belonging to a positively charged extension of the DNA-binding domain, which has been discovered in many DNA-binding proteins (23).

**Figure 8:**
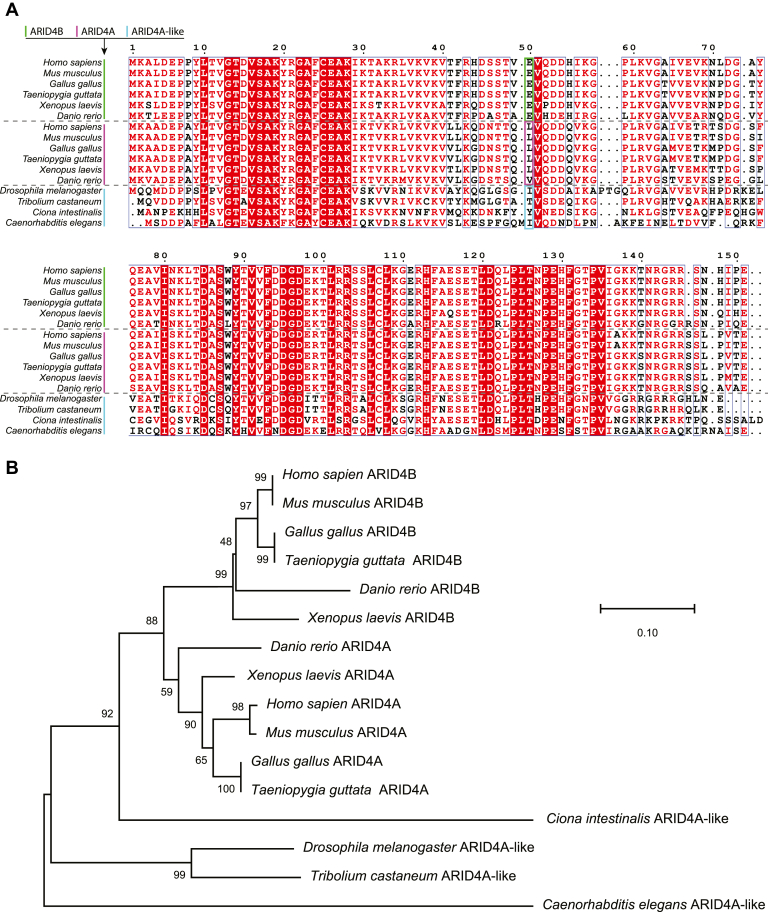
**Bioinformatic analysis of Tudor domain sequences in ARID4A/ARID4B homologue proteins.***A*, sequence alignment. The sequences are indicated by *vertical lines* in *green*, *magenta*, and *cyan* for ARID4B, ARID4A, and ARID4A-like proteins with the species name. *B*, phylogenetic analysis conducted using MEGA X (36). The evolutionary history is inferred using the neighbor-joining method. The percentage of replicate trees in which the associated taxa clustered together in the bootstrap test (1000 replicates) are shown next to the branches.

Sequence alignment and phylogenetic analysis of the Tudor domains and the C-terminal extensions of the ARID4A/ARID4B family proteins revealed different conservation of the Glu50/Leu50 residues and the RGR motif (Fig. 8). Higher animals from *Danio rerio* to *Homo sapiens* contain both ARID4A and ARID4B homologues, while lower animals contain only one homologue. In higher animals, all ARID4B Tudor domains contain the Glu residue at the corresponding position to Glu50, while all ARID4A Tudor domains contain a Leu/Val residue at the same position. The corresponding residues in the homologues of lower animals show more variation without charge, which is more similar to ARID4A than ARID4B (Fig. 8*A*). Therefore, Glu50 of ARID4B has likely evolved for the specific function of ARID4B after it diverged from an ARID4A-like ancestor (Fig. 8*B*), implying its importance for the function of ARID4B. Our observation of different DNA-binding affinity and stability caused by the difference between Glu50 and Leu50 could be related to the specific functions of the two homologous proteins. The RGR motif is largely conserved in both ARID4A and ARID4B in higher animals, but less conserved in the ARID4A-like homologues of lower animals. Therefore, the function of the RGR motif is likely to be important for both ARID4A and ARID4B in higher animals, but less important in lower animals. The results presented in this study not only provide novel molecular insight into the functional differences between the homologous ARID4A and ARID4B proteins, but also shed light on the role and importance of the RGR intrinsically disordered region in the ARID4A/ARID4B protein family.

## Experimental procedures

### Protein expression and purification

The ARID4B constructs include TD121 (residues 1–121), mutant E50L of TD121, TD151 (residues 1–151), and mutants (K139A, R142A, G143A, R144A and R145A of TD151). The ARID4A constructs include TD121 (residues 4–121), TD151 (residues 4–151), and mutant L50E of ARID4A TD121. The proteins were constructed in pET28a with an N-terminal His_6_-SMT3 tag and then expressed and purified as described previously for other His-SMT3-tagged proteins (24). ^15^N-^13^C-labeled ARID4B TD121 and TD151 were prepared using the same procedures, except that cells were grown in M9 minimal medium containing ^15^NH_4_Cl and [^13^C]-glucose as the sole nitrogen and carbon sources.

### NMR spectroscopy

All NMR experiments on ARID4B TD151 to obtain NMR assignments and distance restraints were performed at 302 K on Bruker AVANCE 600 MHz or 800 MHz spectrometers, each of which was equipped with a triple-resonance cryoprobe. NMR samples of ARID4B TD151 contained 0.6 mM protein in buffer A (20 mM Na_2_HPO_4_-NaH_2_PO_4_, 100 mM NaCl, pH7.0), with addition of 5 mM DTT, 0.02% (w/v) sodium 2,2-dimethylsilapentane-5-sulfonate (DSS), and 10% (v/v) D_2_O. The two-dimensional ^1^H-^15^N and ^1^H-^13^C heteronuclear single quantum coherence (HSQC) and three-dimensional CBCA(CO)NH, HNCACB, HNCO, HN(CA)CO, HBHA(CO)NH, HCCH-TOCSY, CCH-TOCSY experiments were performed for backbone and side chain assignments of ARID4B TD151. The three-dimensional ^1^H-^15^N and ^1^H-^13^C NOESY-HSQC spectra with mixing times of 120 ms were collected to generate distance restraints. All data were processed using NMRPipe (25) and analyzed using NMRViewJ (26). Proton chemical shifts were referenced to the internal DSS, and ^15^N and ^13^C chemical shifts were referenced indirectly.

### Structure calculations

The ARID4B TD151 structure was initially calculated using the program CYANA (27), and then refined using CNS (28) with semiautomated NOE assignments by SANE (29). Backbone dihedral angle restraints obtained using CSI 3.0 (19) and TALOS-N (30), as well as hydrogen-bond restraints according to the regular secondary structure patterns, were also used in the structural calculation. From 200 CNS-calculated conformers, 50 lowest-energy conformers were selected for further water refinement using CNS and RECOORDScript (31). The resulting 20 energy-minimized conformers were used to represent the solution structure of ARID4B TD151. The quality of the determined structure (Table 1) was analyzed using PROCHECK-NMR (32) and MolMol (33). Structural figures were created with MolMol (33) and PyMOL (34).

### DNA titration

DNA duplexes used in the titration experiments were 12-mer dsDNA2 and 18-mer dsDNA1 (15), 18-mer A-tract (dsA-tract), 18-mer AT-rich (dsAT18), and 18-mer GC-repeat (dsGC18). Sequences of these DNAs are as follows: dsDNA2, 5'-CTG TCA AAG GTG-3' (forward), 3'-AC AGT TTC CAC T-5' (backward); dsDNA1, 5'-CTC AGG TCA AAG GTC ACG-3' (forward), 3'-AG TCC AGT TTC CAG TGC T-5' (backward) dsA-tract, 5'-CGC TTT AAA AAA TTT CGG-3' (forward), 3'-GCG AAA TTT TTT AAA GCC-5' (backward); dsAT18, 5'-CGC AAT TAT ATA TTA CGG-3' (forward), 3'-GCG TTA ATA TAT AAT GCC-5' (backward); dsGC18, 5'-CGC ACC GAT CCG TGA CGG-3' (forward), 3'-GCG TGG CTA GGC ACT GCC-5' (backward). Double-stranded DNA was made by annealing equimolar amounts of the two synthesized single-stranded DNAs (1:1 M ratio), which were dissolved in a buffer containing 50 mM Tris-HCl (pH 7.6) and 50 mM NaCl (buffer A), heated to 94 °C for 3 min, and then cooled slowly to room temperature. DNAs were further purified by gel filtration and then concentrated. The stock solution contained 5 mM DNA duplexes in buffer A. ARID4B TD121 and TD151 protein samples were extensively dialyzed against buffer A before the titration.

Interaction of ARID4B TD121 and TD151 with DNA was monitored by recording a series of two-dimensional ^1^H-^15^N HSQC spectra of proteins at each DNA titration point. The observed chemical shift perturbations (CSPs) of the protein resonances were calculated using the equation:

$$CSP=\sqrt{{\left(\delta_{HN}\right)}^{2}-{\left(\frac{\delta_{N}}{5}\right)}^{2}}$$

Where δ_HN_ and δ_N_ are the changes of ^1^H^N^ and ^15^N chemical shifts, respectively. The equilibrium dissociation constants (*K*_D_) of protein with DNA were estimated by fitting the CSPs to the equation:

$$CSP=\frac{CSP_{max}}{2}\left[\left(1+n\times r+n\times K_{D}\left(\frac{1}{C_{pro}}+\frac{r}{C_{lig}}\right)\right)-n\times \sqrt{{\left(1/n+r+K_{D}\left(\frac{1}{C_{pro}}+\frac{r}{C_{lig}}\right)\right)}^{2}-4r/n}\right]$$

where CSP_max_ is the CSP value at the theoretical saturated condition obtained from the titration curve fitting; r is the molar ratio of DNA to protein; C_pro_ is the concentration of initial protein solution; C_lig_ is the stock concentration of DNA. *n* is the number of equivalent and independent binding sites on the DNA, while the physical meaning of the obtained value of *n* is complicated as it could also account for any uncertainty in DNA and protein concentrations that were fixed in fitting. To be consistent with fitting for the ARID4A Tudor domain (15), *n* was fixed as 5 and 3 in the fitting curves of ARID4B TD121 titration with 18-bp dsDNA1 and 12-bp dsDNA2, respectively, and fixed as 3 and 2 for ARID4B TD151 titrated with 18-bp dsDNA1 and 12-bp dsDNA2, respectively.

### Electrophoretic mobility shift assays (EMSAs)

The 12-bp dsDNA2 and 18-bp dsDNA1 were used for EMSA. Free proteins (1.2 mM in 0–8 μl for different ratios of protein *versus* DNA) were mixed with 1 μl dsDNA probe (150 μM), 3 μl glycerol (v/v 40%), and 1 to 9 μl buffer A in a final volume of 13 μl, with 12 μM final dsDNA concentration in each lane. After 60 min incubation at 4 °C, samples were loaded onto 8% native acrylamide gels, run in 1 x TBE buffer (90 mM Tris, 90 mM boric acid, 2 mM EDTA) at 4 °C, 150 V for 30 min. Gels were soaked for 5 to 10 min in TBE buffer containing 0.5 μg/ml ethidium bromide and visualized by TANON 1600 Gel Imager.

### Isothermal titration calorimetry (ITC)

ITC measurements were performed at 25 °C on an iTC-200 calorimeter (MicroCal, Inc). The titrations were carried out in buffer A. The reactant (0.1 mM protein) was placed in the 200-μl sample cell. Then dsDNA solutions in an injection syringe (0.6 mM) were injected into protein solutions in the cell. The volume of each injection was 2 μl except for the first injection, which was 0.4 μl. A titration experiment consisted of 20 consecutive injections of 4 s duration, with a 120 s interval between injections. Control experiments were performed under identical conditions to determine the heat signals that arise from addition of DNA into the buffer. The resulting data were fitted to a single-site binding model using the Origin software package (MicroCal, Inc).

### Thermostability test

Protein samples of ARID4B TD121, ARID4A TD121, and ARID4A L50E mutant, each containing 0.25 mM proteins in 50 μl volume, were incubated for 2 h at 30 °C in the buffer containing 50 mM Tris-HCl (pH 7.5) and 50 mM NaCl. Samples were then centrifuged at 13,000*g* for 30 min. The absorbance of the supernatant at 280 nm was then measured to determine the concentration.

### Differential scanning calorimetry (DSC) experiments

DSC measurements were performed using a Nano DSC system (TA). Prior to scanning, samples were degassed under vacuum for 15 min using a degassing station (TA). DSC thermograms were determined by monitoring the difference in heat capacity in solution upon increasing temperature at a scan rate of 1 °C min^−1^ by heating the sample from 15 °C to 75 °C under increased pressure (3 atm). All proteins used in this study were extensively dialyzed against a buffer containing 50 mM NaCl, 50 mM Tris, pH 7.6, and the dialysis buffer was used for instrumental baseline scans and as reference samples. Protein concentrations used were 1.0 mg/ml, corresponding to 75.0 μM for ARID4A/ARID4B TD121 proteins. Data were fitted to a two-state scaled model using NanoAnalyze software.

### HADDOCK modeling

Structure modeling of the ARID4B TD151 and DNA complex was performed using HADDOCK (22). The starting structural coordinate files for HADDOCK were generated from the 20 conformers of the ARID4B TD151 solution structure and B form dsDNA1 duplex built using the Web 3DNA server (35). For HADDOCK calculations, active residues for ARID4B TD151 were defined as those having weighted CSPs larger than the mean plus standard deviation in the dsDNA1 titration. As residues within the long loop between Gly110 and Lys138 show minor CSP values and significantly affect the docking process, we performed haddock docking with dsDNA1 duplex separately for residues 1 to 110 and 138 to 151 of ARID4B TD151. Residues 1 to 8 and 147 to 151 were also deleted after initial docking as they are not important for DNA binding and their flexibility could lead to steric hindrance during the docking process. Docking of residues 9 to 110 with DNA was performed using the HADDOCK2.2 webserver (22). Passive residues were automatically defined around the active residues by HADDOCK. The active residues were optimized according to the initial docking result, and the final active residues included Lys19-Gly22, Lys33, Lys37, Gln52, Trp88, and Lys99-Ser105. All the bases of the dsDNA1 sequence were considered active in the initial docking. Bases 4 to 8, 10 to 14, and 24 to 28 of dsDNA1 were defined as active residues at final docking, and passive residues of dsDNA1 were automatically defined. A total of 1000 initial structures of the complex were generated for rigid-body docking, and the 200 lowest-energy structures were further refined in explicit water after semiflexible simulated annealing. A cluster analysis was performed on the final 200 water-refined structures based on a 0.6 Å RMSD cutoff criterion. The clusters were ranked based on the averaged HADDOCK score of their top ten structures. The structure in the cluster with the lowest HADDOCK score was selected to represent the model of the ARID4B Tudor domain and dsDNA1 complex.

Docking of the RGR motif, 138-KKTNRGRRS-146, with DNA was performed using HADDOCK2.2 on a local machine. All peptide residues were considered as active during docking. All DNA bases were considered active in the initial docking, and the active residues were then optimized according to the initial docking result. The final active residues included bases 8 to 12 and 25 to 28. As the short peptide sequence is not suitable for clustering, we chose ten structures with the lowest haddock scores from 200 final water-refined structures to represent the model of the complex between DNA and the peptide.

A structure model of the ARID4B TD151 and dsDNA1 complex was constructed by manually combining the models of the Tudor domain-dsDNA1 complex and the RGR motif-dsDNA1 complex. The distance and orientation between the Tudor domain and RGR motif in this model are arbitrarily chosen without further optimization.

## Data availability

All atom assignments of ARID4B TD151 have been deposited in BMRB under accession number 50612. The structure and the restraints have been deposited in the Protein Data Bank under accession number 7DM4 for ARID4B TD151. All remaining data are contained within the article.

## Supporting information

This article contains supporting information.

## Conflict of interest

The authors declare that they have no conflicts of interest with the contents of this article.
