# Correction: Structural basis for the DNA-binding activity of human ARID4B Tudor domain

**DOI:** 10.1016/j.jbc.2022.102492

**Published:** 2022-09-30

**Authors:** Jie Ren, Hongwei Yao, Wanhui Hu, Sarah Perrett, Weibin Gong, Yingang Feng

The authors report a correction of the first equation on page 12 (in the Experimental procedures, DNA titration section). The “minus” operator should be “plus” in the equation as it shown on the following picture.

**Figure:**
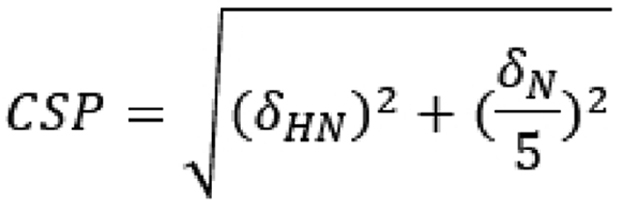

The authors state that the correction does not affect the results and conclusions of the paper.

