# Supplementary material for: Structural basis for the DNA-binding activity of human ARID4B Tudor domain
Source: J Biol Chem. 2021 Mar 4;296:100506. doi: 10.1016/j.jbc.2021.100506 (PMC8038949; doi:10.1016/j.jbc.2021.100506)
Supplement: Supplemental Figures S1–S4 and Table S1 [file mmc1.pdf]

Supporting information

Structural basis for the DNA-binding activity of human ARID4B Tudor domain

Jie Ren (任洁)[1, 5, †], Hongwei Yao (姚宏伟)[2, †], Wanhui Hu (胡万辉) [1,5, #], Sarah Perrett[1, 5], Weibin Gong (宫维斌)[1, *], Yingang Feng (冯银刚)[3,4,5, *],

[1] National Laboratory of Biomacromolecules, CAS Center for Excellence in Biomacromolecules, Institute of Biophysics, Chinese Academy of Sciences, Beijing 100101, China

[2] Institute of Molecular Enzymology, School of Biology and Basic Medical Sciences, Soochow University, Suzhou 215123, China

[3] CAS Key Laboratory of Biofuels, Qingdao Institute of Bioenergy and Bioprocess Technology, Chinese Academy of Sciences, Qingdao 266101, China

[4] Shandong Provincial Key Laboratory of Synthetic Biology, Qingdao Institute of Bioenergy and Bioprocess Technology, Chinese Academy of Sciences, Qingdao 266101, China

[5] University of Chinese Academy of Sciences, 19A Yuquan Road, Shijingshan District, Beijing 100049, China

[#]Current addresses: H. Wu, Shanghai Henlius Biotech, Inc., Building C, 1289 Yishan Road, Xuhui District, Shanghai 200233, China

[†]These authors contributed equally to this work

*To whom correspondence should be addressed: Weibin Gong, E-mail: gongweibin@ibp.ac.cn; Yingang Feng, E-mail: fengyg@qibebt.ac.cn

Table S1. HADDOCK structure calculation statistics for the ARID4B Tudor domain-dsDNA1 model structures of top 7 clusters.

Cluster[a]	Haddock score[b]	RMSD[c]	N[d]	E_{vdw}[e]	E_{elec}[f]	E_{AIR}[g]	BSA[h]	E_{desolv}[i]	*Z-Score*
1	-88 ± 5	2.0 ± 1.2	16	-57 ± 5	-198 ± 15	35 ± 19	1422 ± 161	5.9 ± 2.5	-1.0
3	-84 ± 3	3.7 ± 2.5	14	-50 ± 5	-232 ± 23	60 ± 22	1391 ± 78	6.3 ± 2.5	-0.8
4	-84 ± 18	3.0 ± 1.8	6	-62 ± 10	-185 ± 52	72 ± 10	1594 ± 239	7.5 ± 2.5	-0.8
5	-80 ± 11	2.9 ± 1.8	6	-57 ± 11	-193 ± 34	80 ± 47	1327 ± 193	7.3 ± 3.1	-0.5
7	-80 ± 20	1.5 ± 0.9	5	-64 ± 13	-155 ± 85	86 ± 30	1569 ± 158	6.9 ± 5.7	-0.5
2	-79 ± 8	1.9 ± 1.2	15	-50 ± 2	-220 ± 39	90 ± 44	1374 ± 48	5.3 ± 1.8	-0.4
6	-74 ± 8	2.9 ± 1.2	6	-53 ± 6	-194 ± 50	71 ± 24	1299 ± 93	9.9 ± 3.3	-0.1

[a]The final 200 structures were clustered based on a pair-wise RMSD matrix using a 0.6 Å cutoff. The statistics are for the four lowest energy structures.
[b]The HADDOCK score was calculated as the sum of: $E_{vdw} + 0.2 * E_{elec} + 0.1 * E_{AIR} + E_{desolv}$.
[c]Backbone RMSD of structures in a given cluster.
[d]Number of structures in a given cluster.
[e]van der Waals intermolecular energy.
[f]Electrostatic intermolecular energy.
[g]HADDOCK ambiguous interaction restraint energy (kcal mol^{-1}).
[h]Buried surface area ($Å^2$).
[i]Desolvation energy (kcal mol^{-1}).

Figure S1.

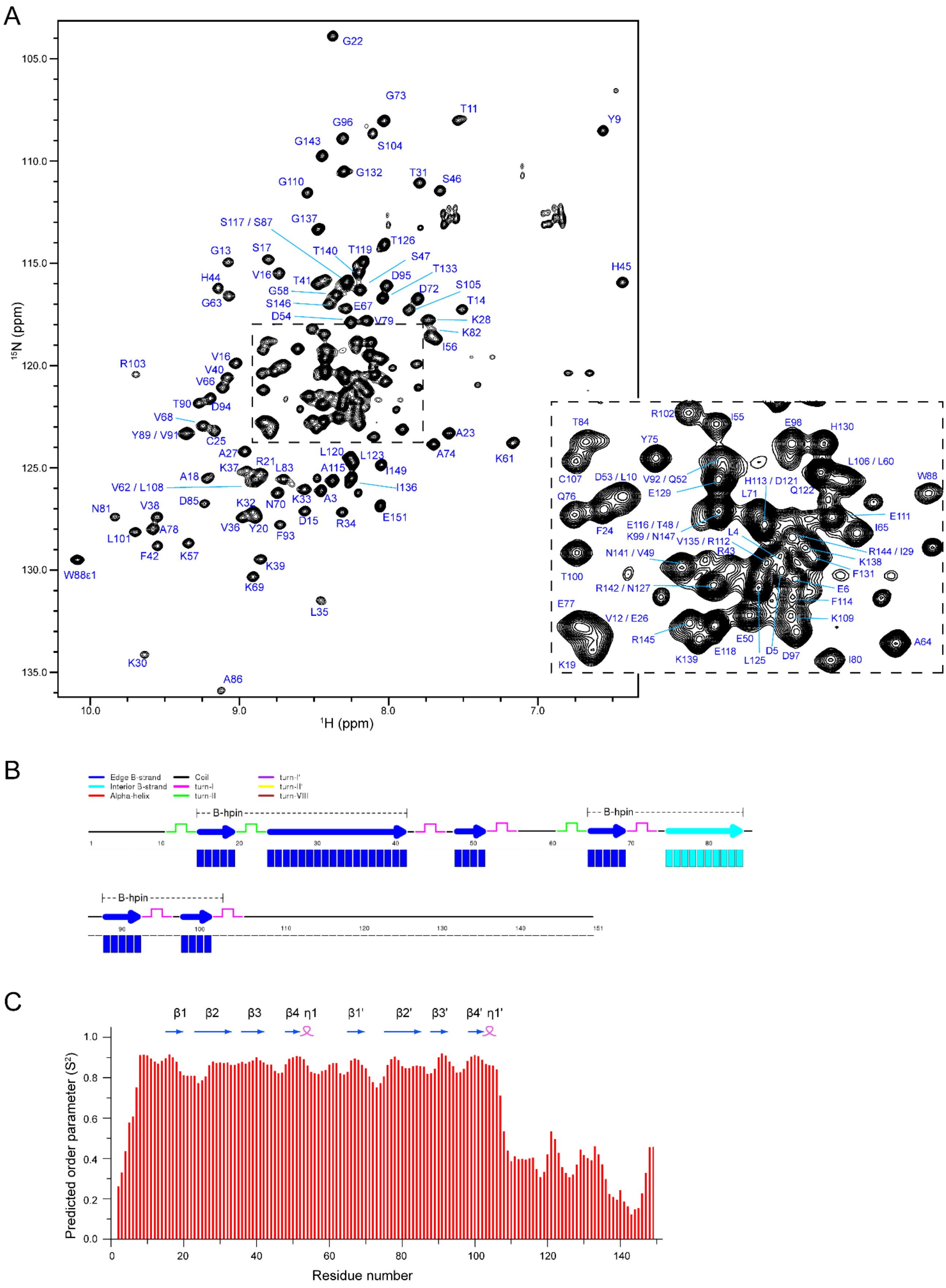
A
^{15}N (ppm)
^{1}H (ppm)
B
Edge B-strand
Interior B-strand
Alpha-helix
Coil
turn-I
turn-II
turn-I'
turn-II'
turn-VIII
B-hpin
C
β1 β2 β3 β4 η1 β1' β2' β3' β4' η1'
Predicted order parameter (S²)
Residue number

Figure S1. Resonance assignments of ARID4B TD151 and predictions of secondary structure and order parameters based on backbone assignments. (A) The ^{1}H-^{15}N HSQC spectrum with NH signals assigned and labelled with one-letter amino acid code and residue number in the sequence. (B) Secondary structure prediction by CSI 3.0 (25). (C) Histogram of predicted order parameters (S^2) of ARID4B TD151 versus residue number by TALOS-N (26). The region with S^2 less than 0.6 is considered to be a highly dynamic/disordered region.

Figure S2.

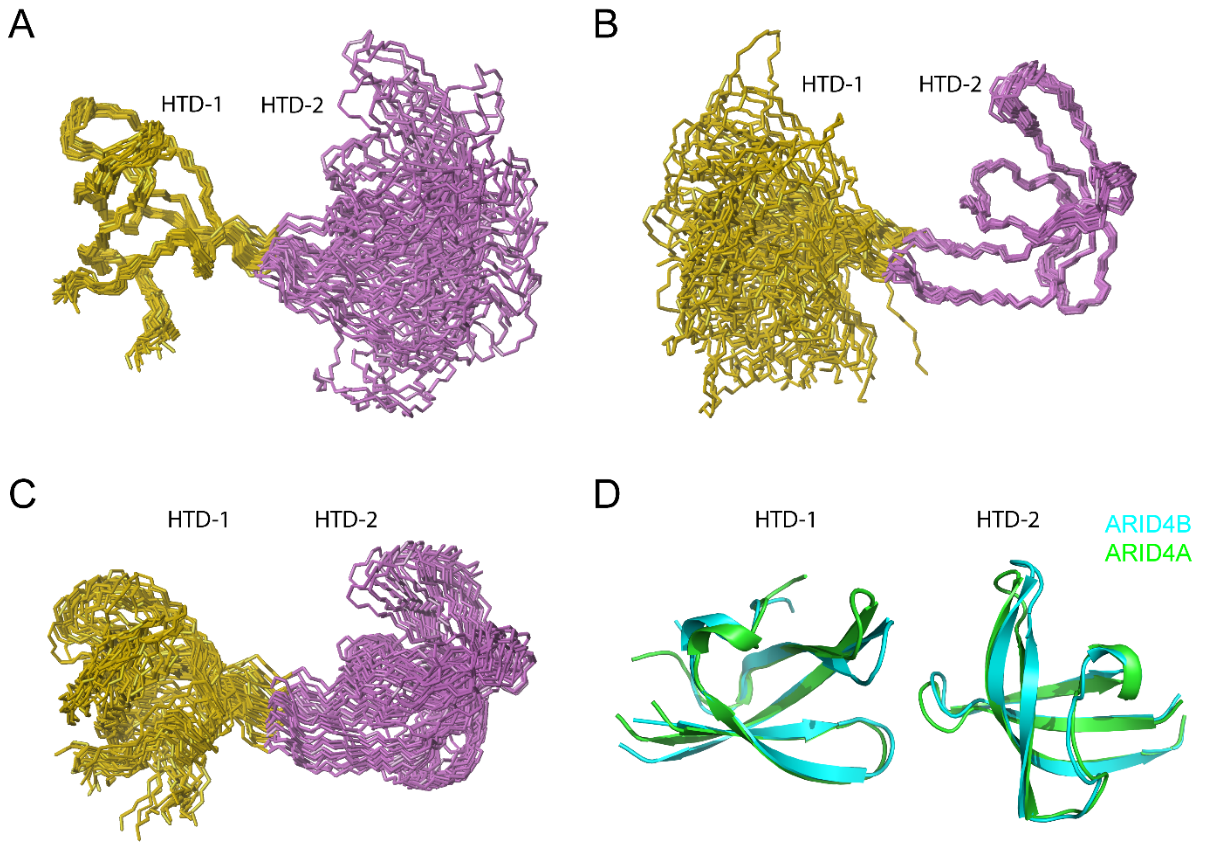

Figure S2. Solution structure of the ARID4B Tudor domain. (A-C) Ensemble of the top 20 lowest energy structures of the ARID4B Tudor domain superimposed on HTD-1 (yellow) (A), HTD-2 (magenta) (B), or on all secondary structure regions (C). (D) Alignments of HTD-1 and HTD-2 subdomains of ARID4A and ARID4B Tudor domains.

Figure S3.

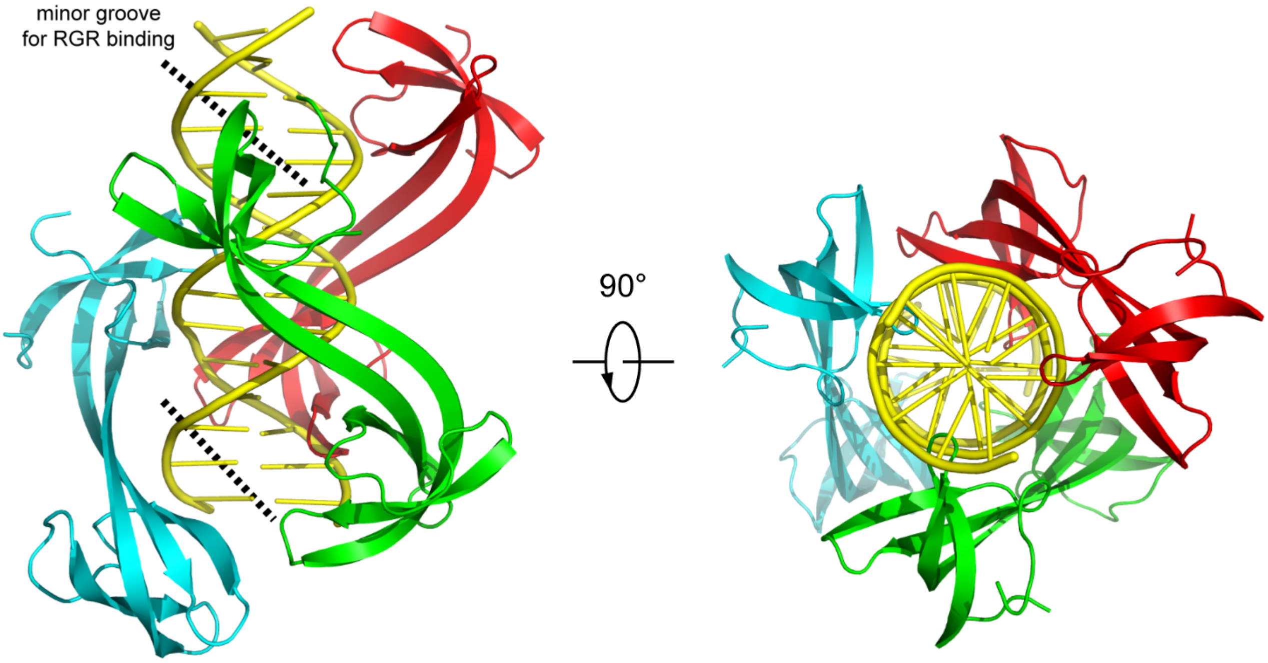

Figure S3. Three ARID4B TD151 molecules can be accommodated on dsDNA1. ARID4B Tudor domain molecules are in cyan, green, and red, and dsDNA1 is in yellow. The RGR motif can bind to the available minor groove indicated by dashed lines at both 5′ and 3′ ends of dsDNA1.

Figure S4.

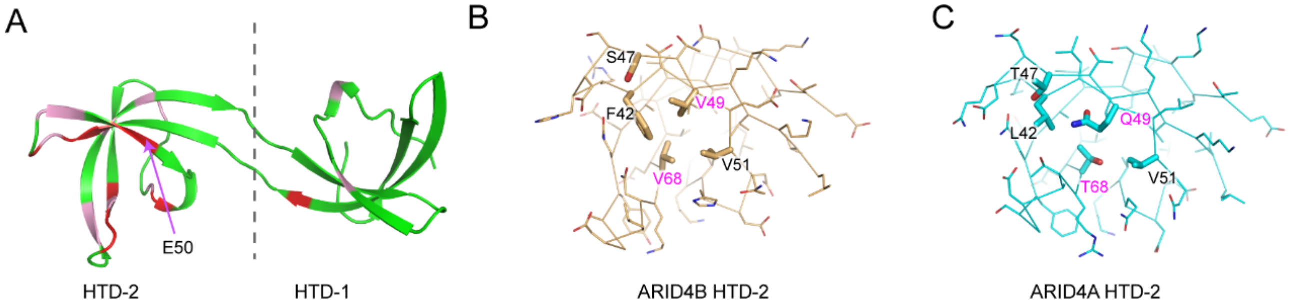

Figure S4. Differences between the HTD-2 subdomains of ARID4B and ARID4A Tudor domains. (A) Mapping of sequence difference (residues 1-110) between ARID4B and ARID4A Tudor domains. Red, residues between ARID4B and ARID4A with no or weak similarity (green residues in Figure 1A). Pink, residues with high similarity. Green, identical residues. (B, C) Comparison of hydrophobic core residues in ARID4B HTD-2 (B) and ARID4A HTD-2 (C).
